# A B2 SINE insertion in the *Comt1* gene (*Comt1*^*B2i*^) results in an overexpressing, behavior modifying allele present in classical inbred mouse strains

**DOI:** 10.1111/j.1601-183X.2010.00614.x

**Published:** 2010-11

**Authors:** R L Kember, C Fernandes, E M Tunbridge, L Liu, J L Payá-Cano, M J Parsons, L C Schalkwyk

**Affiliations:** † Social, Genetic and Developmental Psychiatry Research Centre, Institute of Psychiatry, King's College LondonDe Crespigny Park, London, UK; ‡ Department of Psychiatry, University of OxfordOxford, UK; § Present address: Medical Research Council Mammalian Genome UnitHarwell, UK

**Keywords:** Behavior, comt, genetics, mouse, sine insertion

## Abstract

Catechol-O-methyltransferase (COMT) is a key enzyme for dopamine catabolism and *COMT* is a candidate gene for human psychiatric disorders. In mouse it is located on chromosome 16 in a large genomic region of extremely low variation among the classical inbred strains, with no confirmed single nucleotide polymorphisms (SNPs) between strains C57BL/6J and DBA/2J within a 600-kB window. We found a B2 SINE in the 3′ untranslated region (UTR) of *Comt1* which is present in C57BL/6J (*Comt1*^*B2i*^) and other strains including 129 (multiple sublines), but is not found in DBA/2J (*Comt1*^+^) and many other strains including wild-derived *Mus domesticus, M. musculus, M. molossinus*, *M.castaneus* and *M. spretus. Comt1*^*B2i*^ is absent in strains closely related to C57BL/6, such as C57L and C57BR, indicating that it was polymorphic in the cross that gave rise to these strains. The strain distribution of *Comt1*^*B2i*^ indicates a likely origin of the allele in the parental Lathrop stock. A stringent association test, using 670 highly outbred mice (Boulder Heterogeneous Stock), indicates that this insertion allele may be responsible for a difference in behavior related to exploration. Gene expression differences at the mRNA and enzyme activity level (1.7-fold relative to wild type) indicate a mechanism for this behavioral effect. Taken together, these findings show that *Comt1*^*B2i*^ (a B2 SINE insertion) results in a relatively modest difference in *Comt1* expression and enzyme activity (comparable to the human Val-Met polymorphism) which has a demonstrable behavioral phenotype across a variety of outbred genetic backgrounds.

Catechol-O-methyltransferase (COMT) plays a regulatory role in catecholamine neurotransmission, particularly in the case of dopamine, by facilitating degradation (Tunbridge *et al.* 2004). Dopamine is known to play a role in reward-seeking behavior, cognition and motor activity (Goldman-Rakic *et al.* 2000; Schultz 2001; Yang *et al.* 2003). COMT is therefore an attractive candidate molecule for involvement in these processes.

The most frequently examined human polymorphism in *COMT* is a Valine to Methionine substitution. It has been ascertained that the Val^158^Met SNP results in a change in enzyme activity, but not mRNA levels, with Val^158^ homozygotes having approximately 1.4-fold greater COMT activity than Met^158^ homozygotes in the prefrontal cortex (Chen *et al.* 2004). COMT has been associated, with varying degrees of robustness, to a number of disorders, including schizophrenia (Shifman *et al.* 2002) and obsessive compulsive disorder (Pooley *et al.* 2007), and is also of interest in research into cognition (Egan *et al.* 2001; Tunbridge *et al.* 2006) and aggression (Rujescu *et al.* 2003).

The homologous mouse gene, previously called *Comt*, has been recently renamed *Comt1*. Research in mice has shown a link between *Comt1* expression and cognitive (Papaleo *et al.* 2008) and aggressive phenotypes (Fernandes *et al.* 2004; Filipenko *et al.* 2001; Gogos *et al.* 1998). In *Comt1* knockout mice a variety of phenotypic changes have been reported, including increased anxiety (Gogos *et al.* 1998), improved working memory, set-shifting performance and greater acoustic startle reactivity (Papaleo *et al.* 2008) and lower weight and greater motor activity (Haasio *et al.* 2003). An exploratory and habituation phenotype characterized by increased sifting and chewing has also been found in the mice heterozygous for the *Comt1* deletion (Babovic *et al.* 2007). Mice overexpressing *Comt1* also display a mild phenotype, being less active in the open field but showing no differences in prepulse inhibition (PPI) of the startle response (Stark *et al.* 2009).

In mouse, *Comt1* is situated on chromosome 16, in an area with very little genetic variation between inbred mouse strains (Yang *et al.* 2007). However, a *Comt1* expression difference in the nucleus accumbens and striatum between strains has been noted, with consistently higher expression in the C57BL/6J when compared to the DBA/2J mouse, except for probe sets at the far 3′ untranslated region (UTR) (Grice *et al.* 2007; Korostynski *et al.* 2006). An outbred highly recombinant mouse stock is an optimal way to stringently test mice for a phenotypic effect resulting from genotypic or expression differences (Chia *et al.* 2005). The Boulder Heterogeneous Stock (HS) mice (McClearn & Hofer 1999) were generated from 8 inbred progenitor strains and now have over 65 generations of accumulated recombination, creating a highly variable genetic background on which to examine phenotype. This allows phenotypic differences between the progenitor strains (including C57BL/6J and DBA/2J) to be associated with genetic loci.

We have identified a polymorphism in *Comt1* that mediates the expression difference observed between strains and provides a possible resolution for the conflicting expression data between different probe sets. Additionally, we examine whether this *Comt1* polymorphism is associated with a behavioral phenotype using the HS mouse stock.

## Materials and methods

### Animals

DNAs from 44 different inbred strains of mice were purchased from the Jackson Laboratory (Bar Harbor, ME, USA; http://www.jax.org/dnares/). Male C57BL/6J and DBA/2J animals used to prepare hippocampal extracts for COMT1 activity assays were bred in the SPF facility at the Institute of Psychiatry, London, from original stocks obtained from the Jackson Laboratory via Charles River UK. Male, HS (McClearn & Hofer 1999) mice, excluding albinos, were obtained from the Institute for Behavioral Genetics, University of Colorado at Boulder (Boulder, USA) and shipped to the UK in 8 batches (80–100 mice per batch) at the age of approximately 8 weeks. The average age of HS mice at the start of open field testing was 90.24 ± 2.92 days (mean ± SD), and all HS mice were from generations 64–72. DNA was prepared from spleens of male C57BL/6J, DBA/2J and HS mice. RNA was prepared from hippocampi of male C57BL/6J and DBA/2J mice. The hippocampus was chosen as the source for the mRNA for this study as this is a key area of the brain involved in behaviors such as learning and memory, anxiety and aggression (Fernandes *et al.* 2004). All animal works were licensed under the Animals (Scientific Procedures) Act 1986, reviewed by the ethical review panel of the Institute of Psychiatry and the Home Office inspectorate, and are in accordance with the European Communities Council Directive of 24 November 1986 (86/609/EEC).

### Tissue collection

Animals were killed by cervical dislocation and were immediately dissected and tissues snap frozen and stored at −80°C until use.

### Primers, sequencing and genotyping

**Figure 1:**
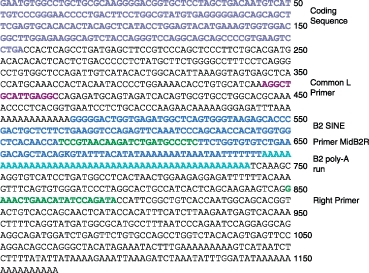
**Annotated sequence for the C57BL/6J strain *Comt1* gene**. Sequence is a composite of RefSeq cDNA sequence NM_001111062 and sequence of the B2 insertion found in this study. Sequencing of additional strains shows identical sequence in those with the insertion, except for some differences in the length of the poly-A run at the end of the B2 element. Primers used for the sequencing and genotyping are displayed in color, as is the position of the B2 SINE insertion in the Comt1^*B2i*^ allele.

Primers shown in Fig. 1 were designed based on the RefSeq cDNA sequence NM_001111062, which is a composite of partial cDNA sequences of strain C57BL/6 origin, and were used to amplify a region of between 239 and 475 bp. PCR reactions contained 20 ng of template, 0.33 µm of each primer, 200 µm each nucleotide, 50 mm KCl, 0.1% Tween-20, 1.5 mm MgCl_2_, 35 mm Tris base and 15 mm Tris–HCl in a final volume of 30 µl, using a touchdown protocol with annealing temperature beginning at 55°C and stepping down to 50°C. PCR products were sequenced after cleanup with an Exo-SAP kit (USB Corporation, Staufen, Germany). The B2 insertion was identified using RepeatMasker (http://www.repeatmasker.org).

Primer pair 5′-AGGCTGCATTGAGGC-3′ (Common L primer) and 5′-GAAACTGAACATATCCAGATA-3′ (Right primer) were used to assay a panel of inbred strain DNAs and 670 HS DNAs for *Comt1*^*B2i*^ by agarose gel electrophoresis. To ensure correct calling of heterozygote genotype we used an additional PCR reaction with primers 5′-AGGCTGCATTGAGGC-3′ (Common L primer) and 5′-TCCGTAACAAGATCTGATGCCCTC-3′ (MidB2R, in B2 sequence) to assay the presence or absence of *Comt1*^*B2i*^.

### Gene expression

Profiles of the hippocampi of 265 of the HS male mice were determined using the Affymetrix GeneChip® Mouse Exon 1.0 ST Array (Santa Clara, CA, USA) in experiments which will be fully described elsewhere. Briefly, RNA was extracted using TRIzol reagent (Invitrogen, Paisley, UK) and labeled and hybridized using the Affymetrix WT synthesis and labeling system according to the manufacturer's recommended protocols. The resulting data were normalized and summarized using the RMA sketch method of the Affymetrix power tools and further quality controlled and analyzed using the R packages ‘Affy’ (Gautier *et al.* 2004) and ‘Exonmap’ (Okoniewski *et al.* 2007).

### 3′ RACE

Length of the 3′ UTR of *Comt1* mRNA in C57BL/6J mice was determined using the Invitrogen 3′ RACE system for rapid amplification of cDNA ends. Briefly, RNA was extracted using the Qiagen AllPrep DNA/RNA mini kit (Crawley, UK). Using the 3′ RACE kit, cDNA was synthesized according to the manufacturer's instructions. A ‘one-sided’ PCR was performed using 5′-AGGCTGCATTGAGGC-3′ (Common L primer) and a universal amplification primer that binds to the 3′ end of the cDNA. Length of the resulting PCR product was determined using agarose gel electrophoresis. All visible bands were extracted using Qiagen QIAquick gel extraction kit (Crawley, UK) and sequenced.

### Enzyme assay

COMT1 enzyme activity was assayed in crude protein extracts as previously described (Tunbridge *et al.* 2007). Briefly, tissue was thawed on ice and homogenized in 25 mm Tris pH 7.4, 50% v/v glycerol and protease inhibitors (Complete Protease Inhibitor Cocktail Tablets, Roche, Burgess Hill, UK). Fifty micrograms of total protein was incubated at 37°C for 30 min in 100 mm Tris pH 7.4, 5 mm MgCl_2_, 100 mm catechol and 2 mm dithiothreitol, supplemented with 3.6 µCi per reaction of ^3^H-S-adenosylmethionine (specific activity: 5–15 Ci/mmol; Perkin Elmer, Waltham, MA, USA). Reactions were stopped with 1 volume of 1 n HCl and tritiated methylated catechol was extracted by mixing thoroughly with Monoflow 1 scintillation fluid (National Diagnostics, Atlanta, GA, USA). Samples were measured using a liquid scintillation counter. Each data point is the mean of four replicates, the individual results of which were highly correlated (*R* values between 0.955 and 0.992). Specific activity is expressed as counts per minute incorporated in 30 min/50 ug protein with background values (obtained by assaying protein extraction buffer only) subtracted.

### Behavior

A comprehensive behavioral battery was conducted on males from a number of inbred strains as well as BXD and HS mice (Galsworthy *et al.* 2005, 2002; Lad *et al.* 2007, 2009). This large-scale experiment included 670 HS mice. The battery, which is described in detail in Lad *et al.* (2009), included eight behavioral tests: activity monitoring in the home-cage (1st and 23rd hour after transfer to a fresh cage); open field; novel object exploration; elevated plus-maze; light/dark box; puzzle box; Morris water maze; tail suspension test.

### Statistical analysis

From our battery of eight behavior tests (Lad *et al.* 2009), we selected 54 measures for association testing with *Comt1*^*B2i*^. For details of the behavioral measures used for association and their selection see Lad *et al.* (2009). Association was tested by one-way analysis of variance (anova) with genotype as a categorical variable, implemented using the lm() function of *R*. Multiple testing was addressed using the false discovery rate approach (Storey & Tibshirani 2003).

## Results

### Novel Comt1 allele

**Figure 2:**
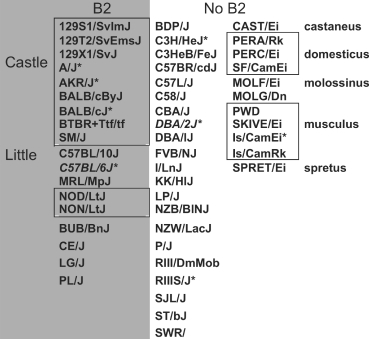
**Strain distribution**. Presence of *Comt1*^*B2i*^ across inbred strains. Boxes group related strains, asterisks denote HS stock progenitors (or in the case of Is/CamEi and RIIIS/J, the probable nearest surviving relative).

No confirmed SNPs were identified in a survey of the *Comt1* exons in 12 strains (A/J, AKR/J, BALB/cJ, C3H/HeJ, C57BL/10J, C57BL/6J, DBA/2J, ISCamEi, ISCamRK, RIIIDmMob, RIIIS/J and PWD/Ph). A length polymorphism of nearly 200 bp was identified in the 3′ UTR of *Comt1* between C57BL/6J and DBA/2J, possessing the long and the short alleles, respectively. Sequence analysis of the PCR product from the above 12 inbred strains and two additional strains (NOD/LtJ and NON/LtJ) indicated that the length difference was because of a single insertion, consistent with the C57BL/6J sequence (RefSeq NM_001111062), found to be present in seven of the strains sequenced. RepeatMasker showed that the length difference is because of an insertion of a B2 SINE of family 1t (Jurka *et al.* 2005; Smit 2005; Smit *et al.* 1996–2004). The consensus sequence (Smit 2005) lacks 2 G residues at the 5′ end of our sequence and there are further probable discrepancies in the AT-rich 3′ portion. Excluding these regions, BLAT searching of the core 153 bp of the insertion (which deviates from the B2_Mm1t consensus at five positions) identifies a single perfect match which is at positions 136082980–136083132 on Mm 5, which would be a candidate parent for this insertion. By inspection the likely insertion site would be ATTT/A and the target site duplication would consist of a run of 15 As. We have named this allele *Comt1*^*B2i*^. Therefore, the presence of *Comt1*^*B2i*^ was surveyed in the strains listed in Fig. 2.

### Gene expression

**Figure 3:**
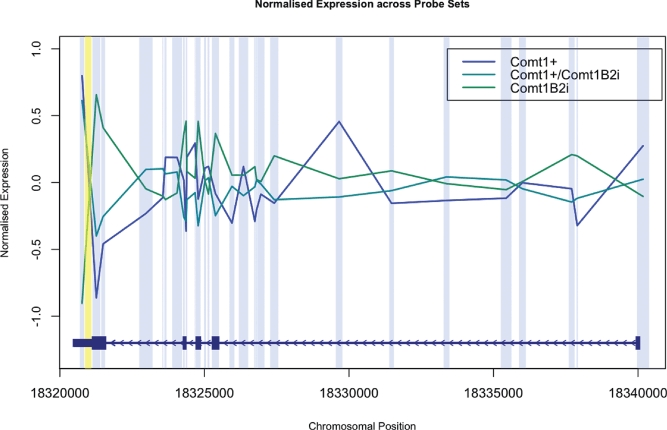
**Exon array *Comt1* gene expression data**. Genotype group means of standardized array signal intensities are plotted by genomic position. *Comt1*^+/+^ expression means are plotted in dark blue, *Comt1*^+*/B2i*^ in teal and *Comt1*^*B2i/B2i*^ in green. The structure of the gene is shown at the bottom, in genomic orientation (the 3′ end is at the left). Probe set positions are marked in light blue. The position of the B2 SINE insertion is shown in yellow. Probe sets flank, but do not span, the insertion site. Data presented are based on NCBI36/mm8.

Hippocampus consortium data (Overall *et al.* 2009; http://www.genenetwork.org), produced using the Affymetrix MOE430v2 array, shows very strong *cis* expression QTL (eQTL) signals for probe sets 1449183_at (LOD 7.3) and 1418701_at (LOD 30), but with opposite directions of effect. *Comt1*^*B2i*^ is associated with increased expression for probeset 1449183_at, whereas the *Comt1*^+^ allele is associated with increased expression for probe set 1418701_at. A replication in the outbred HS animals, using a different array platform and population, showed an additive effect of genotype on expression. Genotype group means of standardized array signal intensities across the *Comt1* gene are shown in Fig. 3.

### 3′ RACE

PCR of the 3′ UTR of Comt1 mRNA in C57BL/6J produced a band of approximate length 300 bp. Preliminary sequencing of the product indicates that the 3′ UTR includes the B2 SINE. Furthermore, the 3′ UTR ends at the 3′ end of the B2 insertion.

### Enzyme activity

**Figure 4:**
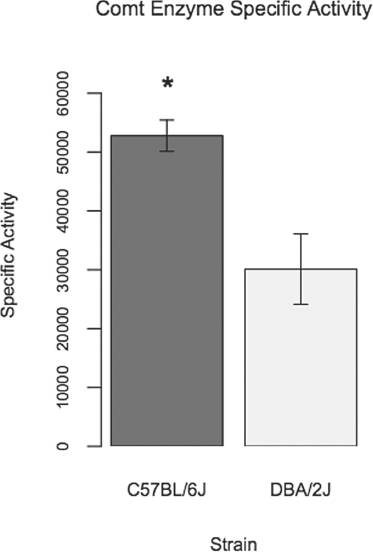
**Comt enzyme activity**. Means ± standard error of the mean for specific Comt enzyme activity. Enzyme activity is around 1.7-fold higher in C57BL/6J (*Comt1*^*B2i*^) compared to DBA/2J (*Comt1*^+^) (*t* = 3.43, *df* = 14, *P* = 8 × 10^−3^).

COMT1 activity was assayed in hippocampal protein from adult male C57BL/6J and DBA/2J mice. The specific activity of COMT1 in C57BL/6J hippocampus shows a 1.7-fold difference compared to DBA/2J (*t* = 3.43, *df* = 14, *P* = 0.008, two-tailed test, Fig. 4).

### Outbred behavior and genotyping

We surveyed *Comt1*^*B2i*^ and behavior of 670 male mice from the outbred HS population (McClearn & Hofer 1999). The progenitors of this population are eight inbred strains (A, C57BL/6, BALB/c, AKR, DBA, C3H, Is/Bi and RIII), and all these are represented in Fig. 2 except for the last two, of which the probable nearest surviving relative is shown. Four of the eight progenitor strains are thus known to be *Comt1*^*B2i*^ and therefore substantial numbers of each allele should be present in the population. We found 53 homozygotes for *Comt1*^+^, 333 *Comt1*^+^*/Comt1*^*B2i*^ and 284 *Comt1*^*B2i*^ homozygotes, giving an allele frequency of 0.672 for *Comt1*^*B2i*^ in the whole population.

**Figure 5:**
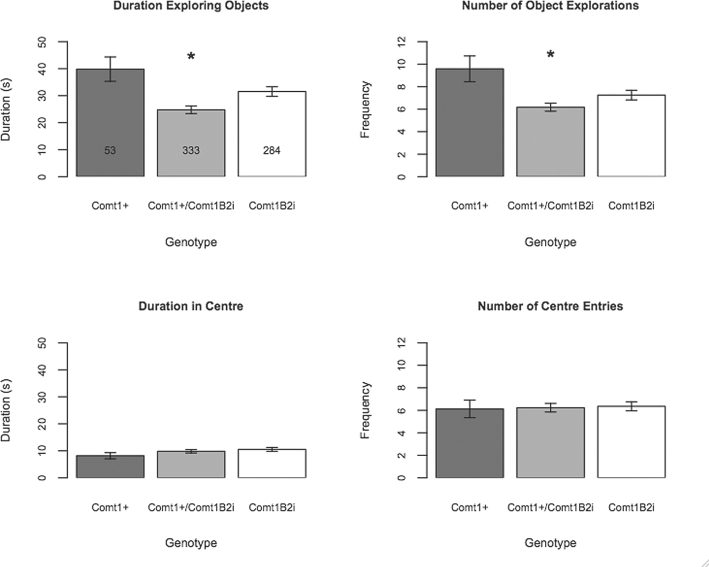
**Behavior**. Means ± standard error of the mean for measures from the novel object task (NO) and the open field task (OF) in the HS mice grouped by *Comt1*^*B2i*^ genotype. Duration and frequency of exploration in the novel object are greater in *Comt1*^+^ than the other genotype classes, and least in the *Comt1*^+^/*Comt1*^*B2i*^ genotype (Duration: *F*_2,572_ = 8.7, *P* = 1 × 10^−4^; Frequency: *F*_2,572_ = 6.1, *P* = 2 × 10^−3^). No significant differences are seen in the OF.

The novel object exploration test was the only behavioral measure to show a significant association with *Comt1*^*B2i*^ (Duration: *F*_2,572_ = 8.7, *P* = 1 × 10^−4^; Frequency: *F*_2,572_ =6.1, *P* = 2 × 10^−3^, Fig. 5). Both the duration and the frequency of exploration of the novel object were greater in *Comt1*^+^ compared to the *Comt1*^+^/*Comt1*^*B2i*^ and *Comt1*^*B2i*^ genotype groups. The *Comt1*^*B2i*^ is therefore dominant, at least with respect to the behavioral phenotype. There were no differences in anxiety measures of the open field test, performed on the previous day in the same arena, or in any of the other behavioral tasks in our battery.

## Discussion

We have found that a B2 SINE in *Comt1* (*Comt1*^*B2i*^) present in some inbred strains but not others is the likely cause of the expression difference between these strains, being the only confirmed variation within the gene between C57BL/6J and DBA/2J. *Comt1*^*B2i*^ is associated with an increase in specific enzyme activity, as well as changes in behavior related to exploration. These findings suggest that a modest difference in *Comt1* expression levels can have a significant behavioral phenotype, in line with previous findings (Fernandes *et al.* 2004).

Sequencing indicates that the insertion site and the B2 SINE insert sequence are identical across *Comt1*^*B2i*^ strains, except for possible length variation in the flanking poly A runs, suggesting identical origin of *Comt1*^*B2i*^. Comparison of the strain distribution of *Comt1*^*B2i*^ with what is known of the breeding history of the inbred strains (Beck *et al.* 2000) makes it immediately evident that although C57BL/6J is *Comt1*^*B2i*^, several very closely related strains (C57L, C57BR) are not. Therefore, *Comt1* must have been polymorphic in the cross between Miss Lathrop's female 57 and male 52 that gave rise to these strains (Beck *et al.* 2000). *Comt1*^*B2i*^ is not present in our sampling of wild-derived *Mus domesticus, M. musculus, M. molossinus* and *M. castaneus* strains. Those classical inbred strains that do contain the insertion are almost all known to have ancestry from the Castle and Little stocks, suggesting that *Comt1*^*B2i*^ actually arose in the Lathrop stock around the start of the 20th century. The exception is the pair of strains NOD/LtJ and NON/LtJ, which are of ‘Swiss’ origin, without a known connection to Castle's stocks.

The frequency of insertional mutagenesis by SINEs in mouse is unknown, but at least one similar case has been reported in the literature: *Alas1* contains a B2 SINE insertion in DBA/2J but not C57BL/6J (Chernova *et al.* 2008). Research into the most common human SINE, Alu, hypothesizes a retrotransposition rate of 1 new insertion per 20 births (Cordaux & Batzer 2009). Additionally, transposition is much more active in the mouse genome when compared to the human genome, with transposons being found to be responsible for about 10% of spontaneous mutations (Guenet 2005).

Using Affymetrix exon array data, we showed in the hippocampi of the HS that *Comt1*^*B2i*^ is associated with high gene expression signal for probes 5′ of the insertion site but low expression for 3′ located probes. The opposite relationship is seen in *Comt1*^+^ mice, while *Comt1*^+^/*Comt1*^*B2i*^ mice have intermediate expression at these loci. Microarray analysis using the Affymetrix MG_U74Av2 microarray with a single *Comt1* probe set (98535_at) found variation between eight strains in the hippocampus (Fernandes *et al.* 2004). C57BL/6J had the highest *Comt*1 expression and DBA/2J the lowest, and this difference correlated with an aggressive phenotype. Subsequent microarray studies showed similar strain differences in *Comt1* expression in the nucleus accumbens (Grice *et al.* 2007) and striatum (Korostynski *et al.* 2006). In the latter study it was noted that a probe set located further 3′ in the gene showed a strain difference in the opposite direction. This relationship is also clearly visible in hippocampus across the BXD recombinant inbred panel (Overall *et al.* 2009; http://www.webQTL.org) which shows strong (Mendelian) *cis*-genetic effect for the probe set 1418701_at (DBA/2J allele increasing expression) and probe set 1449183_at (C57BL/6J allele increasing expression). One possible explanation for the expression difference is that the B2 insertion may lead to a new polyadenylation site resulting in a modified 3′ UTR. A 3′ RACE conducted on C57BL/6J mRNA shows a transcript ending at the 3′ end of the B2 insertion, 460 bp shorter than the reference sequence (NM_ 001111062). Polyadenylation of the transcript occurs at the polyadenylation signal (AATAAA) contained with the B2 sequence. No evidence was found for a longer 3′ UTR in strains bearing the insertion, suggesting that the shorter 3′ UTR is the dominant transcript. In animals with the insertion, the shorter 3′ end could result in no signal from the furthest out probe sets, accounting for the low signal seen in *Comt1*^*B2i*^ mice.

Based on our current data and that of previous studies it is clear that there is a difference in transcript structure and abundance between inbred strains with *Comt1*^+^ compared to *Comt1*^*B2i*^. We have showed that this polymorphism is associated with functional consequences (Fig. 4) as hippocampal COMT1 enzyme activity is substantially greater in C57BL/6J (*Comt1*^*B2i*^) than in DBA/2J (*Comt1*^+^). This may be mediated to some extent by the presence of a shorter 3′ UTR in *Comt1*^*B2i*^. This difference in enzyme activity is particularly worth noting in comparison to the most studied polymorphism in humans, the Val/Met, which produces a 1.4-fold difference in protein activity with significant behavioral differences. As the difference we found is at a magnitude of around 1.7-fold, we would expect to see similar levels of behavioral differences in an outbred population of mice.

Given the key role played by COMT1 in dopamine metabolism, we tested whether *Comt1*^*B2i*^ might have a behavioral effect. We used HS outbred mice to perform a stringent association test, in which the effect of *Comt1*^*B2i*^ locus is examined against a large panel of different highly heterozygous genetic backgrounds, generated by over 65 generations of accumulated recombination from eight inbred progenitor strains (Boulder Heterogeneous Stock, McClearn & Hofer 1999). We investigated the behavioral phenotypes of the HS mice using a test battery (Lad *et al.* 2009) which includes tests of baseline activity, and measures relating to anxiety, depression and cognition. The majority of these measures did not show an effect of *Comt1*^*B2i*^; however, there was an association with novel object exploration. *Comt1*^*B2i/B2i*^and *Comt1*^+*/B2i*^ mice spent less time exploring, and made fewer visits to a novel object than do *Comt1*^+/+^ mice. *Comt1*^*B2i*^ does not seem to alter anxiety levels as no differences were seen in any of the classical anxiety tasks used in the battery (open field, elevated plus-maze or light/dark box). However, we cannot definitively exclude an anxiety effect as the novel object task was performed in a potentially aversive environment, given that mice had only one previous exposure to the open field and may not have fully habituated to the novel arena. Further experiments testing novel object exploration in a familiar (home-cage) environment could be used to address this issue.

Behavioral differences were to be expected in the light of previous research, where several transgenic *Comt1* mice have been engineered. A knockout of the gene produces a remarkably mild phenotype in terms of basic behavior (Babovic *et al.* 2007; Gogos *et al.* 1998), although associations with cognitive phenotypes are more robust (Papaleo *et al.* 2008). More recently, strains overexpressing *Comt1* have been produced by bacterial artificial chromosome (B AC) transgenesis (Stark *et al.* 2009) and transgenic overexpression of the higher activity Val allele of the heavily studied Val^158^Met human polymorphism (Papaleo *et al.* 2008). In the Stark *et al.* study, PPI was the main phenotype of interest and no effect of *Comt1* overexpression was noted, although these findings are inconsistent with those of Papaleo *et al.* (2008) who showed a reduction in PPI in *COMT-Val-tg* overexpressors and an increase in acoustic startle reactivity, but no change in PPI in *Comt1* knockout mice, compared with their respective wild-types. Additionally, open field was studied and a small difference in total distance traveled noted. Papaleo *et al.* also found impairments in several cognitive measures, including attentional set shifting and working memory in *COMT-Val-tg* overexpressors, compared with wild-type controls, although these mice were created on a mixed genetic background.

Given the results obtained in the *Comt1 and COMT-Val-tg* transgenic mice, it is perhaps surprising that we found associations only with object exploration. Impaired emotional reactivity was observed in *Comt1* knockout mice (Gogos *et al.* 1998) but this effect was only seen in female mice and the present study used males. However, the behavioral effect we have observed is no doubt just part of the phenotype attributable to *Comt1*^*B2i*^, but without more specific phenotyping of higher cognitive functions and behavioral analysis in both male and female mice, our results should be considered preliminary. Given our previous observation of a correlation across inbred strains between *Comt1* expression with intermale aggression, it would be of interest to test these mice in the resident-intruder paradigm. Furthermore, the results of Papaleo *et al.* (2008), and the associations between cognitive function and COMT in humans, suggest that it would be worthwhile to investigate the phenotype of these mice in detailed cognitive tasks. However, given that cognitive tests are generally labour-intensive, and the high-stress nature of the resident-intruder paradigm, studies of this type require specific testing, rather than as part of a test battery as used here.

Our data taken together provide evidence that the *Comt1*^*B2i*^ is itself linked to the changes in object exploration. The association of *Comt1*^*B2i*^ with decreased exploration holds across a large panel of outbred genetic backgrounds. If the responsible allele were at another locus, even one quite closely linked to *Comt1*, it is likely that recombination would have occurred between the causative locus and the *Comt1* gene, and that the association with *Comt1*^*B2i*^ would therefore have been lost. Furthermore, our survey of the exons of the *Comt1* gene showed no other confirmed polymorphisms in a set of strains that represent as far as possible the progenitors of the *Comt1* locus, which is in an extensive region of identity-by-descent across the classical inbred strains (Yang *et al.* 2007). Between C57BL/6J and DBA/2J, there is no known polymorphism within the *Comt1* gene.

The molecular mechanism for the altered COMT1 enzyme activity remains unclear. Given the complexity of the microarray results, it is unlikely to be because of a simple difference in mRNA abundance. The longer insertion-bearing transcript is polyadenylated at the 3′ end of the insertion, resulting in a shorter 3′ UTR. This may lead to alternate processing by miRNAs, or may result in an altered ratio of COMT1 protein isoforms. Other instances of insertional mutagenesis by retrotransposition have been associated with physical phenotypes (Duhl *et al.* 1994; Ho *et al.* 2004), and the mechanism for these events is not always clear. Further dissection of the effects of this allele, both in terms of behavioral phenotype and molecular biology, will be informative about the normal function of the *Comt1* gene.
